# Supplementary material for: Birth order dependent growth cone segregation determines synaptic layer identity in the Drosophila visual system
Source: eLife. 2016 Mar 17;5:e13715. doi: 10.7554/eLife.13715 (PMC4846375; doi:10.7554/eLife.13715)
Supplement: Supplementary file 1. — The table shows detailed genotypes used in each of the experiments shown in figures and arranged to depict genotypes analysed for each representative image in the figures. DOI: http://dx.doi.org/10.7554/eLife.13715.022 [file elife-13715-supp1.docx]

**Supplementary Table 1- List of Genotypes used in this study.**

| Figure 1 A | *ey3.5 FLP/+; FRT42 UAS-mCD8GFP/FRT42 Gal80; Sens-Gal4 UAS-mCD8GFP/+* |
| --- | --- |
| Figure 1 C, D, E | *+/+; +/+; Sens-Gal4/UAS-mCD8GFP* |
| Figure 1 C’, D’, E’ | *+/+; sev-Gal4/CyO; UAS-mCD8GFP/TM2* |
| Figure 2 A, A’ | *hs-FLP/+; FRT42 UAS-mCD8GFP/FRT42 Gal80; sens-Gal4 UAS-mCD8GFP/+* |
| Figure 2 B, B’ | *hs-FLP/+; FRT42 sequoia^5^/FRT42 Gal80; sens-Gal4 UAS-mCD8GFP/+* |
| Figure 2 C, C’, E, E’ | *GMR-FLP/+; FRT42 UAS-mCD8GFP/FRT42 Gal80; PM181-Gal4 UAS-mCD8GFP/+* |
| Figure 2 D, D’, F, F’, G, G’ | *GMR-FLP/+; FRT42 sequoia^5^/FRT42 Gal80; PM181-Gal4 UAS-mCD8GFP/+* |
| Figure 2 H, H’ | *GMR-FLP/+; FRT42 UAS-mCD8GFP/FRT42 Gal80; LGMR-Gal4 UAS-mCD8GFP/TM2* |
| Figure 2 I, I’ | *GMR-FLP/+; FRT42 sequoia^5^/FRT42 Gal80; LGMR-Gal4 UAS-mCD8GFP/TM2* |
| Figure 2 J, J’ | *GMR-FLP/GMR-gogo; FRT42 UAS-mCD8GFP/FRT42 Gal80; LGMR-Gal4 UAS-mCD8GFP/TM2* |
| Figure 2 K, K’ | *GMR-FLP/GMR-gogo; FRT42 sequoia^5^/FRT42 Gal80; LGMR-Gal4 UAS-mCD8GFP/TM2* |
| Figure 3 A, A’ | *+/+; sev-Gal4/CyO; UAS-mCD8GFP/TM2* |
| Figure 3 B, B’ | *+/+; sev-Gal4/UAS-Sequoia; UAS-mCD8GFP/TM2* |
| Figure 3 C, C’ | *GMR-FLP/+; FRT42 UAS-mCD8GFP/FRT42 Gal80; LGMR-Gal4 UAS-mCD8GFP/TM2* |
| Figure 3 D, D’ | *GMR-FLP/+; FRT42 UAS-Sequoia/FRT42 Gal80; LGMR-Gal4 UAS-mCD8GFP/+* |
| Figure 3 E, E’ | *Ey3.5-FLP/+; FRT42 UAS-Sequoia/FRT42 Gal80; sens-Gal4 UAS-mCD8GFP/TM2* |
| Figure 3 G, G’ | *ey3.5 FLP/+; FRT42 /FRT42; ro-τ-LacZ/+* |
| Figure 3 H, H’ | *ey3.5 FLP/+; FRT42 sequoia5/ FRT42; ro-τ-LacZ/+* |
| Figure 3 I, I’ | *ey3.5-FLP/+; FRT42 UAS-mCD8GFP/ FRT42 Gal80; LGMR-Gal4 UAS-mCD8GFP/TM2* |
| Figure 3 J, J’ | *ey3.5-FLP/+; FRT42 UAS-Sequoia/ FRT42 Gal80; sens-Gal4 UAS-mCD8GFP/TM2* |
| Figure 4 A-E | *+/+; UAS-Sequoia/ tub-Gal80ts; LGMR-Gal4/ GMR-GFP* |
| Figure 5 A-D’ | *+/+; +/+; GMR-GFP/GMR-GFP* |
| Figure 5 E-F’ | *ey3.5-FLP/+; sens-Gal4 UAS-mCD8GFP/ CyO; Gal80 FRT2A/FRT2A* |
| Figure 5 G-H’ | *ey3.5-FLP/+; sens-Gal4 UAS-mCD8GFP/ CyO; Gal80 FRT2A/capricious^C18fs^FRT2A* |
| Figure 5 I | *+/+; tub-Gal80ts/ LGMR-Gal4; UAS-capricious^RNAi^/ GMR-GFP* |
| Figure 5 J | *+/+; tub-Gal80ts, UAS-Sequoia/ LGMR-Gal4; GMR-GFP/ TM2* |
| Figure 5 K | *+/+; tub-Gal80ts, UAS-Sequoia/ LGMR-Gal4; UAS-capricious^RNAi^/ GMR-GFP* |
| Figure 6 A, A’, C, C’, E, E’ | *+/+; UAS-Sequoia/ tub-Gal80ts; LGMR-Gal4/ GMR-GFP* |
| Figure 6 B, B’, D, D’, F, F’ | *UAS-Sequoia/ tub-Gal80ts; LGMR-Gal4/ Rh6-EGFP* |
| Figure 6 G, G’ | *ey3.5-FLP/+; FRT42 sequoia^5^/ FRT42 Ubi-GFP^nls^; +/+* |
| Figure 6 H, H’ | *+/+; UAS-Sequoia/ LGMR-Gal4; +/+* |
| Figure 6 I, I’ | *+/+; UAS-Sequoia/ CyO; caps-lacZ^nls^/ TM2* |
| Figure 6 J, J’ | *+/+; UAS-Sequoia/ LGMR-Gal4; caps-lacZ^nls^/ TM2* |
| Figure 6 K-K” | *+/+; OrtC1-3 LexADBD, OrtC2B dVP16AD/ CyO; LGMR-Gal4/ UAS-Syb::spGFP1-10, LexAop spGFP11::CD4* |
| Figure 6 L-L” | *GMR-gogo/sev-Gal4; PanR7Gal4/ OrtC1-3 LexADBD, OrtC2B dVP16AD; UAS-Capricious^ID^/ UAS-Syb::spGFP1-10, LexAop spGFP11::CD4* |
| Figure 6 M-M” | *sev^E1^/y; LGMR-Gal4/ OrtC1-3 LexADBD, OrtC2B dVP16AD; UAS-Sequoia, tub-Gal80ts/ UAS-Syb::spGFP1-10, LexAop spGFP11::CD4* |
| Supplementary Figure 1 A | *CantonS* |
| Supplementary Figure 1 B | *+/+; sev-Gal4/ CyO; UAS-GFP^nls^/ TM2* |
| Supplementary Figure 1 C, D | *+/+; Bl/CyO; sens-Gal4/ UAS-GFP^nls^* |
| Supplementary Figure 2 A, A’ | *hs-FLP/+; FRT42/ FRT42 Gal80^ts^; sens-Gal4-UAS-CD8GFP/+* |
| Supplementary Figure 2 B, B’ | *hs-FLP/+; FRT42 Sequoia^5^ / FRT42 Gal80^ts^; sens-Gal4-UAS-CD8GFP/+* |
| Supplementary Figure 3 A, A’ | *+/+; +/+; LGMR-Gal4/ UAS-CD8GFP* |
| Supplementary Figure 3 B, B’ | *+/+; UAS-Sequoia/+; LGMR-Gal4/ UAS-CD8GFP* |
| Supplementary Figure 3 C | *CantonS* |
| Supplementary Figure 3 D, D’ | *ey3.5-FLP/+; FRT42 UAS-mCD8GFP/ FRT42 Gal80; m∂0.5 Gal4 UAS-mCD8GFP/ TM2* |
| Supplementary Figure 3 E, E’ | *ey3.5-FLP/+; FRT42 sequoia^5^/ FRT42 Gal80; m∂0.5-Gal4 UAS-mCD8GFP/ TM2* |
| Supplementary Figure 3 F, F’ | *ey3.5-FLP/+; FRT42 UAS-Sequoia/ FRT42 Gal80; m∂0.5 Gal4 UAS-mCD8GFP/ TM2* |
| Supplementary Figure 4 A-D | *+/+; UAS-Sequoia/tub-Gal80ts; LGMR-Gal4/ GMR-GFP* |
| Supplementary Figure 5 A | *+/+; GMR-GFP/ UAS-Sequoia Gal80ts; LGMR-Gal4/+* |
| Supplementary Figure 5 B | *+/+; GMR-GFP/ UAS-Sequoia Gal80ts; LGMR-Gal4/ UAS-Capricious* |
| Supplementary Figure 6a A, A’ | *+/+; OK371-VP16AD/ CyO; ortC2-Gal4DBD/ UAS-mCD8GFP* |
| Supplementary Figure 6a B, B’ | *+/+; UAS-Sequoia/ CyO; LGMR-Gal4/ GMR-GFP* |
| Supplementary Figure 6b A-B’ | *GMR-FLP/+; CadN^405^ FRT40/ Gal80 FRT40; elav-Gal4 UAS-CD8GFP/ TM2* |
| Supplementary Figure 6b C-D’ | *GMR-FLP/+; CadN^405^ FRT40/ Gal80 FRT40; elav-Gal4 UAS-CD8GFP/ UAS-Sequoia* |
| Supplementary Figure 6b E, E’ | *ey3.5-FLP/+; Gal80 FRT40/ FRT40; Sens-Gal4 UAS-CD8GFP/ UAS-Sequoia* |
| Supplementary Figure 6b E, E’ | *ey3.5-FLP/+; CadN^405^ FRT40/ Gal80 FRT40; Sens-Gal4 UAS-CD8GFP/UAS-Sequoia* |
